# Address, Delivered before the Virginia State Dental Association

**Published:** 1872-01

**Authors:** James F. Thompson


### ARTICLE II.

### *Address, Delivered before the Virginia State Dental Association.*

BY JAMES F. THOMPSON, M.D., D.D.S.

*Gentlemen:*—In accordance with our preconcerted plans to inaugurate in a more formal manner the Virginia State Dental Association, we meet to-day in this proud old capital of the mother of States, to lay upon the altar of our Association renewed pledges of professional fealty and respect. It is with much pleasure that I greet you upon this occasion, and beg to congratulate you upon the success that is about to crown the efforts of a determined few, who assembled here one year ago, to advance higher the standard of our profession, which should be the pride of every dentist's heart.

The auspices under which we are permitted to assemble are indeed most cheering. When we look abroad upon this old commonwealth, which but a few years ago lay torn, bleeding and impoverished: to-day stands forth in bright array, full of hope and vigor. She garners in an abundant harvest and she sends forth her hardy yoemen and skillful artisans from every section of her borders to this, her metropolis, to lay upon her common altar the products of their genius and toil, and vieing in honorable competition for a prize, the reward of their labor and skill. We too are here from the office and laboratory, to lay upon the altar of our profession, the products of our experience and skill, and to tune the chord upon which vibrates professional fellowship and love.

Gentlemen, it fills my heart with pride, when I look back and see what rapid strides the dental profession has made within the last four decades of years; proud to see it in the front ranks of the sciences; proud to see it standing close by the side of its twin sister, medicine; but to realize it seems like a dream.

It is needless for me to go into the history of the dental science, for I take it that every educated dentist knows it well. It is needless for me to carry you into the catacombs of Egypt, and there show you specimens pertaining to our art, specimens of a once enlightened age, illuminated by the pen of Heroditus, the father of historians, whose light down through the mediæval ages becoming obscure, it again burst with a blaze of effulgent glory upon the eighteenth century, when the versatile Frenchmen, catching up the inspiration, added new lustre and gave an impetus to dental science, which, wafted across the Atlantic, sounded the key note upon the shores of America; and to-day the American dentist stands second to none. Boasting as we are ever proud to do of the oldest Dental College in the known world, that noble old pioneer, the "alma mater" of many of us here present, the Baltimore College of Dental Surgery, together with others that have sprung and are almost yearly springing into existence throughout the length and breadth of this land, can it therefore, be any wonder why we should not take the lead in our profession? I, gentlemen, am an advocate for the collegiate system as necessary to the thorough professional education of dentists. These institutions are to the dental student what the medical colleges are to the medical student. Let no one here for a moment think that I would reflect disparagingly upon any one of my professional brethren who have never had the advantage of a dental college education. I know of many such, who to-day by their high attainments and skill, would adorn and enoble any profession. To such I am ever ready to extend the hand of fellowship, but why should this continue in a land of dental schools? Many of our sister States have legal enactments, requiring all dental practitioners to be thoroughly educated and proficient, before entering upon the practice of their profession; and shall this noble old State, the mother of State's statesmen, be a laggard in making such an important movement.

Look what the consequence is; her doors are thrown wide open to an influx of many ignorant and unskillful operators, doing such miserable work, imposing upon the credulity of a confiding people, bringing reproach and dragging down a profession whose standard we are here trying to advance. I will ask what do the people as a mass know what the qualifications of a dentist should be?

Take them when their lives are endangered by disease, and see what efforts are made to secure the best medical skill. Take them when their bodies are torn and mangled, and the knife is to be applied, and see with what eagerness they try to secure the best surgical skill.

But, sirs, when teeth are attacked by that insidious enemy caries, and the ravages of decay are doing the sure work of destruction, do they stop and ask for the best dental skill to stay the hand that is working such destruction, maring the human face divine, and bringing disease upon the body? No; they are satisfied to employ the merest ignoramust who calls himself a dentist.

Let us gentlemen, in our associated capacity, give this subject our serious consideration. Let it be our chief aim to advance higher the standard of our profession, to pull down that of the mountebank and quack, and seek to place them under the bands of law.

I for one am opposed to the passage of a law that would in any way be oppressive upon any portion of the community, but the object of one is so manifestly just, that it must commend itself to every thinking mind that would take the trouble to look into it. I feel safe in saying there is no dentist in this State, who is fit to discharge his professional obligations to his patients, but will rejoice in its passage; and I feel safe in making the assertion that the mere fact of any dentist opposing a movement of this kind would be a sufficient cause for doubting his ability.

Gentlemen, I believe with the amount of intelligence we have around us, that the people can be made to become interested in this matter. Let us educate their minds upon the subject and the difficulty is at once removed. Let them go with us to our halls of Legislature, and ask for a law to protect themselves against the ignorant mountebank and quack. It is not sufficient that we as a body of professional men should make such an effort, for fear the question might be raised as to who wants this law and for whose benefit, the dentists or the people. I hope this association will give this, as well as other matters that will be brought before it, a thoughtful consideration. Let us show to the community at large that we are progressive men and are unwilling to hide our light under a bushel.

In the formation of dental associations we at once promote friendly intercourse among dentists and inspire members with professional pride, laudable zeal, and an earnest desire to press forward, to improve and excel in our dental operations. There is no doubt but that the different dental associations throughout the land are doing a noble work.

They have done much to break down the barriers of selfishness, and to place the profession upon a liberal and learned basis, in contrast to those days when dentistry was for the most part a secret art.

But now by the untiring efforts and zeal of a few noble spirits that have passed away, we find art and science most happily blended, and in language most beautifully expressed by a brother of our profession, "That science and art, the twin sisters, who so beautifully and harmoniously blend their characters and attributes in our calling, and ever growing, ever moving, ever adding to the already full stature, and lofty proportions; ever travelling onward and upward toward perfection. The achievements of to-day are but the finger posts that point to the discoveries of to-morrow. The triumphs of to morrow will reveal some hidden truth or develop some richer treasure for its successor."

It is here, gentlemen, in our associated capacity, that we challenge free discussion, free thought and opinion upon whatever subject that pertains to our professional advancement, and that we may render ourselves respectable and respected, useful and be appreciated.

Thanking you gentlemen for your courtesy and kind attention, rest assured that it is my fervent desire that we may all live to be proud that we are members of the Virginia State Dental Association and to see it eminently successful and prosperous, and an ornament to this proud old State whose name it bears.

